# Supplementary material for: Conditional chemoconnectomics (cCCTomics) as a strategy for efficient and conditional targeting of chemical transmission
Source: eLife. 2024 Apr 30;12:RP91927. doi: 10.7554/eLife.91927 (PMC11060718; doi:10.7554/eLife.91927)
Supplement: Figure 3—source data 3. [file elife-91927-fig3-data3.zip › Figure 3 source data3 part 2.zip!/Figure 3-Source data 3-part2/1063-1-CRISPResso_Report_az97gqtx.zip!/CRISPResso_on_1063_3/1a.Read_barplot.pdf]

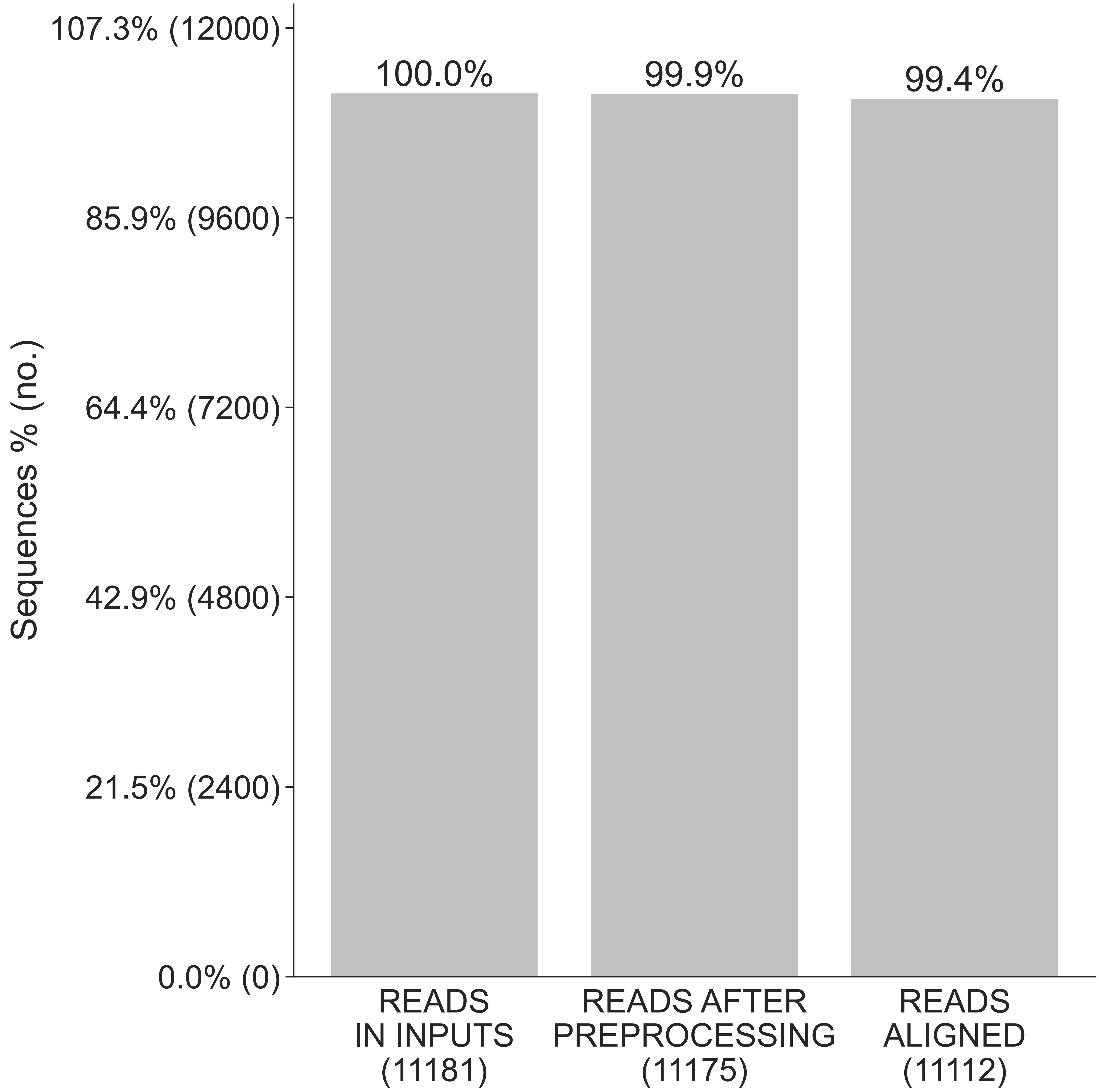
Sequences % (no.)
107.3% (12000)
85.9% (9600)
64.4% (7200)
42.9% (4800)
21.5% (2400)
0.0% (0)
100.0%
99.9%
99.4%
READS IN INPUTS (11181)
READS AFTER PREPROCESSING (11175)
READS ALIGNED (11112)
